# Linking physical activity to personal values: feasibility and acceptability randomized pilot of a behavioral intervention for older adults with osteoarthritis pain

**DOI:** 10.1186/s40814-022-01121-0

**Published:** 2022-08-01

**Authors:** Jennifer C. Plumb Vilardaga, Sarah A. Kelleher, Allison Diachina, Jennie Riley, Tamara J. Somers

**Affiliations:** Pain Prevention and Treatment Research Program, Duke University School of Medicine, 2400 Pratt St., 7th Floor, Durham, NC 27705 USA

**Keywords:** Osteoarthritis, Pain, Physical activity, Values

## Abstract

**Background:**

Osteoarthritis (OA) pain is common and leads to functional impairment for many older adults. Physical activity can improve OA outcomes for older adults, but few are appropriately active. Behavioral interventions can reduce physical activity barriers. We developed and tested a brief, novel behavioral intervention (i.e., Engage-PA) for older adults combining values to enhance motivation and strategic activity pacing to improve arthritis-related pain and functioning and increase physical activity.

**Methods:**

A randomized feasibility and acceptability pilot trial compared Engage-PA to treatment-as-usual plus fitness tracker (TAU+) in *N* = 40 adults age 65+ with OA pain in the knee or hip. Engage-PA involved two 60-min telephone sessions. All participants wore a fitness tracker to collect daily steps throughout the study and completed baseline and post-treatment assessments of secondary outcomes (arthritis-related pain and physical functioning, physical activity, psychological distress, psychological flexibility, and valued living). The impact of COVID-19 on general well-being and physical activity was also assessed. Descriptive statistics were conducted for feasibility and acceptability outcomes. Indicators of improvement in secondary outcomes were examined via change scores from baseline to post-treatment and performing independent samples *t*-tests to assess for between-group differences.

**Results:**

Feasibility was high; 100% accrual, low (5%) attrition, and 100% completion of study sessions. Acceptability was high, with 89% finding the intervention “mostly” or “very” helpful. Engage-PA participants demonstrated improvements in arthritis pain severity (*M*_diff_ = 1.68, *p* = 0.044, 95% CI [− 0.26, 3.62]) and self-reported activity (*M*_diff_ = 0.875, *p* = 0.038, 95% CI [− 1.85, 0.98]) from baseline to post-treatment as compared to TAU+. Due to pandemic-related challenges, there was a high level of missing data (43%) for daily steps, but available data showed no significant change in steps over time or between the groups. COVID-19 added an additional burden to participants, such that 50% were exercising less, 68% were more sedentary, and 72% lost access to spaces and social support to be active.

**Conclusions:**

Engage-PA is a promising brief, novel behavioral intervention with the potential to support older adults in improving arthritis-related pain and functioning and increasing physical activity. The feasibility and acceptability of Engage-PA are particularly notable as most participants reported COVID-19 added more barriers to physical activity.

**Trial registration:**

ClinicalTrials.gov, NCT04490395. Registered on July 29, 2020

## Key messages regarding feasibility

- Uncertainties existed prior to this study about whether older adults with OA would be interested in participating in a brief behavioral intervention (i.e., Engage-PA) to improve pain, functioning, and increase physical activity, as well as whether the intervention components combining personal values and activity pacing would be acceptable to participants.
- Engage-PA demonstrated feasibility and acceptability at a priori established benchmarks for continued study, such that 100% accrual was met, attrition was low (5%), and all participants assigned to Engage-PA condition completed all treatment sessions, and 89% of participants reported being “mostly” or “very” satisfied with the program, and secondary outcomes showed promising indicators of arthritis pain and physical functioning improvement as well as increases in physical activity.
- Engage-PA may be an intervention that helps older adults increase physical activity when barriers are present, given that participants in the current study reported experiencing additional pandemic-specific barriers (e.g., health or finance instability, reduced access and social support for exercising, increased sedentary behavior, reduced physical activity overall) to engaging in physical activity.

## Background

Osteoarthritis (OA) is one of the most common age-related problems facing older adults [1] and is often a progressive, degenerative condition [2]. Treatments such as surgery and medications are available, but some risks associated with these increase with age, such as complications and mortality from problematic (e.g., chronic, high dose) opioid therapy [3]. OA can lead to persistent pain and declining physical functioning for many older adults.

Physical activity is a low-cost, low-burden approach commonly recommended for older adults with OA as a treatment for pain, prevention of disability, and improving outcomes following joint replacement [4–6]. Importantly, physical activity can be conducted safely for even vulnerable older adults [7]. Physical activity is critical for preserving physical functioning in older adults; lack of physical activity contributes to decreased mobility and persistent pain, reducing the likelihood of independent living and exacerbating other common health conditions [8].

Yet, few older adults with OA engage in recommended levels of physical activity, with common barriers including persistent pain and distress [9]. Behavioral interventions can overcome these barriers by teaching skills to cope with pain and distress to allow for regular engagement in physical activity [10, 11]. There is a need for the development and implementation of behavioral approaches that will address these barriers to improve arthritis-related pain and functioning and increase physical activity in this population.

Increasing physical activity, like any health behavior change, involves both motivation for change and specific skills to initiate and sustain the behavior change. Identifying and setting goals based on personal values, or deeply held, personally chosen statements of meaning, and purpose, are one potentially crucial facet of motivation and commitment for health behavior change [12–14]. Acceptance and commitment therapy (ACT [15]) is a behavioral approach that has an explicit, theory-derived focus on personal values [16, 17]. ACT is unique in that its mechanism of change is psychological flexibility, defined as persistence towards one’s stated values even when psychological barriers (i.e., emotional distress, negative thoughts, pain) and other external challenges (i.e., time) arise [18]. There is a growing body of work using these methods, even in brief formats, demonstrating improvements in health behaviors including smoking [19], weight loss [20], and physical activity [21, 22]. Values-based approaches have promise for adults with pain [23], though one study using these methods with adult veterans (many of whom are older adults) with persistent pain only demonstrated benefits on psychological variables and not in physical activity levels [22]. These researchers recommend that values-based behavioral interventions should increase their focus on physical activity promotion [22].

*Activity pacing* is one critical behavioral skill for increasing physical activity that may assist older adults with OA in engaging in physical activity. An activity-rest cycle is a form of activity pacing that is an important component of behavioral symptom management programs (e.g., pain coping skills training [PCST [24]]) for patients with disease-related pain (e.g., cancer [25–29] and arthritis [11, 30, 31]). The activity-rest cycle is rooted in social cognitive theory [32], with a particular focus on increasing self-efficacy to engage in a particular pain management task. This skill encourages sustaining smaller amounts of activity more consistently over time and slowly building up levels of activity and reducing resting periods (vs. overdoing that leads to increased pain and avoidance of activity). Combining personal values and activity pacing has a promising synergy that may be very well suited to helping older adults with OA pain to improve arthritis-related pain and functioning and increase physical activity.

Our team developed such a novel, combined protocol (i.e., Engage) to manage pain, fatigue, and distress for those with advanced cancer, many of whom were older adults, with promising outcomes [33, 34]. Extending this work to patients with OA-related pain, the Engage protocol was adapted to meet the needs of older adults by integrating values and activity pacing for the express purpose of increasing daily steps. The resulting protocol, Engage-PA, also incorporated wearable fitness trackers to allow participants to self-monitor their daily steps.

The primary aim of the current study was to examine the feasibility and acceptability of delivering Engage-PA to older adults with OA pain. Our hypotheses were that Engage-PA would be feasible for future larger-scale studies if (1) we achieved at least 75% accrual of our planned *N* of 40, attrition was less than 20%, and at least 75% of participants completed both study sessions and (2) acceptability of the intervention was shown with at least 80% reporting the intervention mostly or very helpful. Our secondary aim was to examine the changes in arthritis-related pain and functioning, physical activity, psychological distress, psychological flexibility, and valued living before and after patients engaged in the intervention. Given the timing of the study, we also included a measure of COVID-19 impact to assess the patients’ experience with exercise routines, sedentary behaviors, health and illness, or other losses/instability due to the pandemic.

## Methods

### Recruitment and screening

The current study was a randomized pilot feasibility and acceptability trial of older adults (*N* = 40) with a diagnosis of OA in the knee or hip comparing Engage-PA to treatment-as-usual plus fitness tracker control. Participants were recruited from September 2020 through August 2021. Duke University Institutional Review Board provided ethical approval (Pro00105573), and the trial was registered on www.clinicaltrials.gov (NCT04490395).

Potential participants were recruited from several primary care clinics as part of the Duke Health system, across central North Carolina. Potential participants were identified by electronic medical record review based on the inclusion and exclusion criteria. Potential participants were contacted by trained study staff via telephone and/or email summarizing the study information and inviting participants to answer the screening questions either online or over the phone. Interested and eligible participants were invited to engage in consent procedures. Consent was conducted online (via Research Electronic Data Capture [REDCap], a HIPAA-compliant secure online data collection and management tool) or via telephone or mailed paper packets.

#### Eligibility criteria

The inclusion criteria included (1) adults aged 65 or older, (2) diagnosis of OA in the knee and/or hip, (3) English speaking, (4) ability to participate in telephone sessions, (5) ability to ambulate even if assisted by a cane or walker, and (6) rating worst pain and pain interference within the last week as a 3 or greater out of 10. The exclusion criteria included (1) planned surgery (including joint replacement surgery) during the study duration that would affect or limit mobility for more than 3 weeks, (2) major surgery requiring limited mobility within the past 3 months, (3) myocardial infarction within the past 3 months, (4) fall(s) within the past 3 months that led to immediate medical treatment, (5) current enrollment in cardiac rehabilitation, (6) presence of a serious psychiatric condition, (7) reported or suspected moderate cognitive impairment, (8) indication by a medical provider that exercise should only be medically supervised, and (9) presence of other unmanaged medical condition (e.g., hypertension, diabetes, asthma, neurodegenerative condition) that might lead to unsafe participation as outlined in the Physical Activity Readiness Questionnaire Plus (PARQ-2020 [35]; an evidence-based measure for patient-determined safety for engaging in physical activity) subsequently verified by electronic medical record review and/or via communication with patients’ treating medical team.

### Procedures

**Fig. 1:**
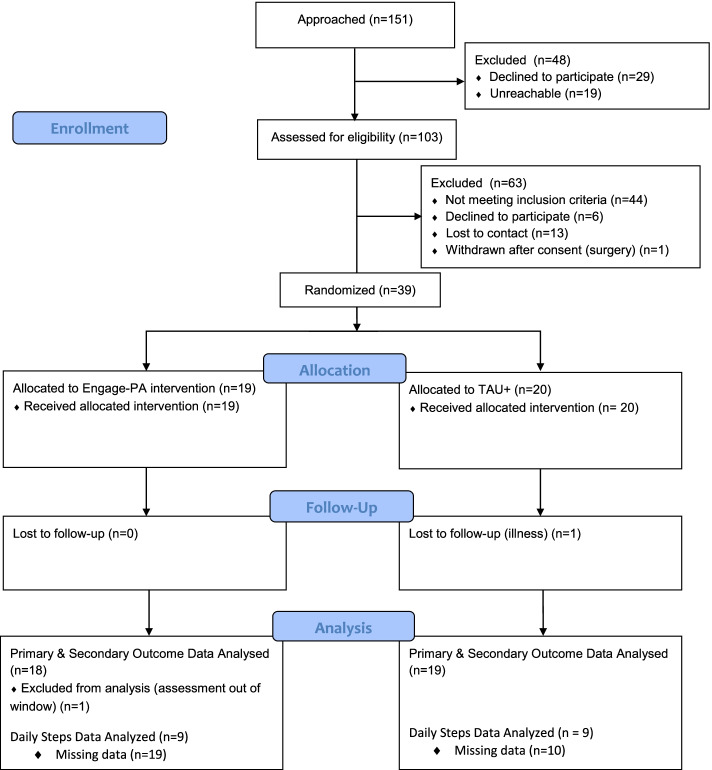
CONSORT flow chart

All procedures were conducted remotely. After consent, participants completed baseline and post-treatment assessments online via REDCap or via telephone or mailed paper packets. All participants received a personal fitness tracker device which provided participants an objective measure of their daily steps. Following completion of baseline assessment, participants were randomized to either active treatment (Engage-PA) condition or treatment-as-usual with fitness tracker (TAU+) control group. Participants in the Engage-PA condition received telephone-delivered treatment sessions. Participants in both groups reported their daily steps as collected by the personal fitness tracker. All participants were compensated $30 for completing each research assessment (baseline and post-treatment) but were not compensated for enrollment or attending intervention sessions. Participation in this study lasted approximately 6 weeks. In Fig. 1, CONSORT diagram provides additional details about participant flow through the study.

#### Allocation to study groups

Randomization was conducted using Randomization Windows Version 5.0 with equal allocation to groups. The study staff who had no contact with study participants conducted randomization; condition assignment was populated in REDCap automatically. Allocation to groups was not concealed from participants. The study staff managing recruitment, assessments, intervention sessions, and fidelity checks were not blinded to condition. Assessment data was entered into REDCap directly by participants wherever possible.

### Study conditions

#### Engage-PA intervention condition

Participants in the intervention group received a study workbook to guide their two 45-min telephone-delivered treatment sessions, spaced approximately 2 weeks apart. To set up later implementation efforts, the intervention sessions were delivered by a master’s level study therapist, guided by a written treatment manual, audio recorded, and checked for fidelity by a senior researcher. In session 1, participants first discussed the fitness tracker and their baseline level of daily steps. The study therapist then explored the participant’s experience with OA pain and physical activity and introduced personal values and activity-rest cycle skills. Personal values were defined as guiding principles that are likely to bring meaning and purpose. Participants were asked to identify two personal values: one related to physical activity and one related to another life domain (e.g., relationships). Personal values were linked to goal setting to allow for more purposeful action, which may increase meaning and satisfaction. Participants were then asked to reflect on their experience with over-doing activity (often resulting in increased pain) or under-doing activity (often resulting in deconditioning) and were subsequently introduced to the activity-rest cycle. The study therapist worked with each participant to identify timed activity bouts and timed strategic rest breaks so as not to exacerbate pain. Participants used the activity-rest cycle to generate specific, timely, and measurable goals related to physical activity (e.g., walk for 10 min) and another value-guided action (e.g., observe a grandchild’s sports event).

In session 2, conducted 2 weeks after session 1, participants reflected on their successes and barriers in doing their specific goals for physical activity and other value-guided activities. The activity rest cycle was troubleshooted in this session, with challenges, successes, and alternative strategies presented. Participants’ specific values were again discussed, and the time was spent considering ways to refine the values and actions to promote success (or increase physical activity). Continuing to engage in a valued activity even if pain or psychological distress increased was highlighted, and the activity-rest cycle was presented as one way to do this.

#### Treatment-as-usual plus (TAU+) condition

The TAU+ condition was characterized by receiving usual care plus a fitness tracker. Usual care in this study meant that participants continued to receive treatment for their arthritis from their current medical teams. Treatments received were those deemed appropriate by their primary care providers (e.g., pain medication) as well as physical therapists (e.g., physical therapy exercises) and orthopedists (e.g., injections) when participants were enrolled in those services. However, upcoming joint replacement surgery was an exclusion criterion for participation. All participants in the TAU+ condition were given a Garmin personal fitness tracker device to self-monitor their steps throughout the study. They also received a handout that they read on their own with instructions on how to use the Garmin device (based on the Garmin user manual) and tips for how to safely engage in physical activity outdoors compiled from the United States National Institutes of Health, National Institute on Aging website (https://www.nia.nih.gov/health/exercising-outdoors).

### Primary outcome measures

#### Feasibility and treatment acceptability

Feasibility was measured by participant enrollment (accrual), attrition, and session attendance. Acceptability was measured by the Client Satisfaction Questionnaire (CSQ) [36, 37] post-treatment. The CSQ is commonly used in treatment acceptability studies and includes items such as “How satisfied are you with the amount of help you received?” and “To what degree did the program teach you skills that are helping you to better manage your symptoms?” rated on a Likert scale.

### Secondary outcome measures

#### Arthritis-related pain and functioning

The Arthritis Impact Measurement Scale (AIMS) [38] was used to assess arthritis pain and functioning. Respondents answer the questions on a 5-point Likert scale where higher scores indicate a greater arthritis-related impact on functioning and health. The symptom subscale includes items of pain frequency, pain severity, sleep interference from pain, and morning stiffness. The physical functioning subscale includes items of arthritis-related mobility, walking and bending, self-care activities of daily living, and household tasks. The AIMS is commonly used to assess functioning related to OA [38].

#### Physical activity (objective and self-report)

Physical activity was measured via both self-report and objective daily step measures.

##### Rapid assessment of physical activity (RAPA) [39]

The RAPA is a 9-item self-report measure where respondents answer a series of yes/no questions about their engagement in a range of aerobic (e.g., light, moderate, vigorous) and non-aerobic (e.g., yoga/flexibility, strength-training) activities, as well as the frequency of their engagement in these activities. Higher scores indicate more activity. The RAPA was designed and validated for use with older adults [39].

##### Daily steps

Participants used a wearable watch-style consumer-grade fitness tracker, a Garmin VivoFit 4.0 ©, upon waking and until bedtime daily throughout the study. Daily step counts were compiled for seven consecutive days at baseline and seven consecutive days post-treatment. Participants either provided daily steps data through online device syncing or a mailed daily step log wherein they wrote down the daily step reading at bedtime from the Garmin device and reported those step counts to study staff via phone or email.

#### Psychological distress

The AIMS [38] affect subscale was used to measure overall psychological distress related to anxiety, tension, and depressed mood. As in the other AIMS subscales, respondents answer the questions on a 5-point Likert scale where higher scores indicate a greater arthritis-related impact on functioning and health.

#### Psychological flexibility

The Acceptance and Action Questionnaire-II (AAQ-II) [40] was used to assess the overall psychological flexibility or the ability to accept difficult psychological experiences as one lives in line with important life goals. In the AAQ-II, participants report their emotional experience (e.g., “I worry about not being able to control my worries and feelings”) on a scale from 1 = “never true” to 7 = “always true.” The AAQ-II has commonly been used as a process of change measure in ACT-based studies and has been related to changes in symptom severity [41, 42].

#### Valued living

The Bull’s Eye Values Survey [43, 44] was used to assess how consistently patients had been living in line with their chosen values. First, patients write about their personal values for living life with meaning and purpose across domains of relationships, education/work/community, leisure, and health/well-being and then rate how successful they have been living in line with these values in the last month. A dartboard image is provided and responses ranged from 1 = “a perfect bull’s eye and great success living in line with a value” to 8 = “very far away from living in line with a value.” This measure has demonstrated utility for pain populations [34] and other behavioral medicine populations [43, 44].

### COVID-19 impact measure

The impact of the COVID-19 pandemic was assessed at baseline for all participants using the COVID-19 Impact Scale (CIS) [45] which was developed and distributed as part of the National Institutes of Health Office of Behavioral and Social Sciences Research toolkit. The CIS was used to assess the overall COVID-19 impact on routines, health, mental health, personal or family member COVID-19 illness/death, and areas of life insecurity (e.g., finances). Additional items were developed for this study, which assessed the changes since the pandemic in the amount of exercise, amount of time spent in sedentary activity, and other ways in which the pandemic affected exercising (e.g., access to fitness centers, social support for exercising).

### Changes to assessments and measurement

Changes to the study assessments and measures were needed after the pilot trial began in response to pandemic-related challenges. First, ActiGraph© accelerometers were initially used as an additional objective measure of physical activity (daily steps, time spent in moderate, vigorous or light/sedentary activity), but due to pandemic-related challenges with remote procedures, two-thirds of the participants did not wear accelerometers. Also due to pandemic-related challenges, Garmin devices were not able to be remotely synced which led to a change in procedures for a subsample of participants (*n* = 27/40). For this subsample, Garmin daily steps data was collected via participant-written daily step logs. Additionally, a third assessment (1 month follow-up) with both self-reported and objectively measured (i.e., daily step count) secondary outcome measures was discontinued after the pilot trial began due to pandemic-related staffing and resource challenges.

### Sample size considerations

In line with the CONSORT guidelines [46], as a pilot feasibility study, a formal power calculation for sample size was not required. The sample size was selected based on previous successful pilot work conducted by our team with similar feasibility pilot goals and planned secondary outcome analyses [33, 34].

### Criteria for success of feasibility and acceptability

A priori criteria for study success were established. We hypothesized that the feasibility of Engage-PA would be met by achieving at least 75% study accrual, a rate of attrition lower than 20%, at least 75% of participants assigned to the Engage-PA condition completing all required study sessions. We hypothesized that treatment acceptability would be met by at least 80% participant satisfaction with the program as measured by the Client Satisfaction Questionnaire.

### Analytic strategy

Primary feasibility and acceptability outcomes were examined using descriptive statistics. For secondary outcomes, paired sample *t*-tests, using a significance value of *p* < 0.05, were calculated to examine the between-group changes (mean difference in change scores) from pre- to post-intervention in arthritis-related pain and functioning, psychological well-being, valued living, and self-reported physical activity. Repeated measures analysis of variance was conducted for baseline and post-assessment daily steps data. Secondary outcome analyses were conducted to examine possible indicators of improvement and given the small sample size should be interpreted with caution [47]. All analyses were conducted using the Statistical Package for the Social Sciences (SPSS©) version 28.

## Results

### Participant characteristics

Demographic information was collected from 39 of the 40 participants (one participant withdrew prior to baseline) and is indicated in Table 1. Participants were aged 65 and older (mean age = 71.77, range 65–90), mostly female (84.6%), with 62% self-identifying as White and 33% self-identifying as Black or African-American. Thirty-seven percent reported an annual household income of less than $60,000/year. The majority of participants had OA in the knee (64%), with 28% reporting OA in both knee and hip. The most common medical co-morbidities reported were hypertension (54%), depression (31%), anxiety (23%), and diabetes (21%).

**Table 1:** Demographic characteristics (*N* = 39)

	***N*** (%)	***M*** (***SD***)
**Age** (years)		71.77 (5.198)
**Gender**		
*Male*	6 (15.4%)	
*Female*	33 (84.6%)	
**Race**		
*Caucasian/White*	24 (61.5%)	
*Black or African-American*	13 (33.3%)	
*2 or more races*	2 (5.1%)	
**Ethnicity**		
*Non-Hispanic*	37 (94.9%)	
*Hispanic or Latino*	2 (5.1%)	
**Education**		
*Less than high school diploma*	1 (2.6%)	
*High school diploma*	2 (5.1%)	
*Some college*	7 (17.9%)	
*Bachelor’s degree*	8 (20.5%)	
*Graduate degree*	21 (53.8%)	
**Income**		
*$10,000 to $19,999*	5 (12.8%)	
*$20,000 to $39,999*	6 (15.4%)	
*$40,000 to $59,999*	2 (5.1%)	
*$60,000 to $100,000*	9 (23.1%)	
*More than $100,000*	13 (33.3%)	

*M* mean, *SD* standard deviation

#### COVID-19 impact

Forty-one percent of participants reported that COVID-19 impacted one or more life areas in a severe way (e.g., finances, social life, mental health, healthcare access, personal or family member COVID illness). In comparison with before the pandemic, 72% reported losing access to locations (e.g., gyms, pools, large indoor walking spaces) or social support for exercising, 68% reported being more sedentary, and 52% of individuals reported exercising less than before the pandemic. Additional participant characteristics are outlined in Table 2.

**Table 2:** Participant medical characteristics and COVID-19 impact (*N* = 39)

	***N*(%)**
**Medical characteristics**	
OA location	
*Knee*	25 (64%)
*Hip*	3 (8%)
*Both*	11 (28%)
Hypertension	21 (54%)
Heart disease	5 (13%)
Rheumatoid arthritis	4 (10%)
Diabetes	8 (21%)
Sciatica	9 (23%)
Emphysema, asthma, or COPD	3 (8%)
Depression	12 (31%)
Anxiety	9 (23%)
Stroke or brain bleed	1 (3%)
Cancer (past or current)	6 (15%)
**COVID-19 impact**	
Reduced access, support for exercise	28 (72%)
Exercising less than prepandemic	20 (52%)
More sedentary than prepandemic	26 (68%)
Same/less sedentary than prepandemic	13 (33%)
Severe COVID impact in 1+ life area	16 (41%)

*OA* osteoarthritis, *COPD* chronic obstructive pulmonary disease, *COVID-19* coronavirus disease

### Feasibility and acceptability results

Feasibility was demonstrated across several metrics. First, all 40 participants (100% accrual) were enrolled within the study time frame. Attrition was low (5%), with only 2 withdrawals: one participant before baseline assessment or randomization (due to surgery) and one between baseline and post-treatment assessment (due to illness). All participants assigned to the Engage-PA intervention condition completed both sessions (100% treatment completion).

Acceptability was high, with 89% of Engage-PA participants finding the intervention “mostly” or “very” helpful. Examples of helpful elements reported were “encouragement,” “setting regular walking goals,” and “clarifying values…to live my best life.” Areas for improvement included suggestions for greater clarity on topics discussed, additional topics to include, and concerns about device use.

### Secondary outcomes

#### Self-report measures

Engage-PA participants demonstrated improvements in arthritis pain (*M*_diff_ = 1.68, *p* = 0.044, 95% CI [− 0.26, 3.62]) from baseline to post-treatment as compared to TAU+. Engage-PA participants also demonstrated improvements in self-reported physical activity (*M*_diff_ = 0.875, *p* = 0.038, 95% CI [− 1.85, 0.98]) from baseline to post-treatment as compared to TAU+ participants. Improvements in arthritis-related physical functioning were approaching significance for the Engage-PA group as compared to TAU+ (*M*_diff_ = 0.875, *p* = 0.056, 95% CI [− 0.15, 1.33]). No other self-reported secondary outcomes were significant. The means for secondary outcome measures by group and over time are presented in Table 3. Self-report secondary outcome results are detailed in Table 4.

**Table 3:** Means for secondary outcome measures by group and time point

	Engage-PA Condition		TAU+ Condition	
	***M***	SD	***M***	SD
Arthritis pain (AIMS symptom subscale)				
Pre	13.72	3.80	14.90	4.16
Post	11.78	3.69	14.58	4.50
Physical functioning (AIMS PF subscale)				
Pre	6.38	1.89	7.44	2.00
Post	6.64	1.38	7.51	2.09
Physical activity (RAPA)				
Pre	3.25	1.22	3.16	1.50
Post	3.47	1.73	2.31	1.19
Total daily steps				
Pre	35,712.56	27,441.79	28,165.89	20,916.05
Post	38,268.56	28,154.67	36,407.78	29,183.00
Psychological distress (AIMS affect subscale)				
Pre	10.36	3.12	10.37	2.85
Post	10.59	4.15	10.33	2.18
Psychological flexibility (AAQ-II)				
Pre	13.95	8.20	16.21	7.45
Post	14.72	8.37	13.05	6.31
Valued living (BEVS)				
*Health domain*				
Pre	4.11	1.99	4.60	1.67
Post	3.76	1.44	4.88	1.73
*Leisure domain*				
Pre	4.26	2.26	4.05	1.99
Post	4.12	1.87	4.18	2.01
*Relationship domain*				
Pre	3.68	2.11	4.00	2.10
Post	4.29	2.29	4.35	2.149
*Work/community domain*				
Pre	3.32	1.97	3.30	1.87
Post	3.65	2.03	3.41	2.24

*Engage-PA* engage protocol, physical activity adaptation intervention group, *TAU+* treatment-as-usual plus fitness tracker control group, *M* mean, *SD* standard deviation, *Pre* baseline assessment time point, *Post* post-treatment assessment time point, *AIMS* Arthritis Impact Measurement Scale, *PF* physical functioning, *RAPA* Rapid Assessment of Physical Activity, *Total daily steps* participant tracked Garmin daily step counts for 7 consecutive days, *AAQ-II* Acceptance and Action Questionnaire, *BEVS* Bull’s Eye Values Survey

**Table 4:** Secondary outcome results for self-report measures

	***M***_**diff**_	***P***	CI (95%), lower, upper
Arthritis pain (AIMS symptom subscale)	1.68	0.044**	− 0.26, 3.62
Physical functioning (AIMS PF subscale)	0.59	0.056*	− 0.15, 1.33
Physical activity (RAPA)	− 0.88	0.038**	− 1.85, 0.98
Psychological distress (AIMS affect subscale)	− 0.24	0.377	− 1.75, 1.28
Psychological flexibility (AAQ-II)	− 2.56	0.073	− 6.05, 0.94
Valued living (BEVS)			
*Health domain*	0.12	0.428	− 1.19, 1.42
*Leisure domain*	− 0.29	0.718	− 1.94, 1.35
*Relationship domain*	− 0.06	0.341	− 0.35, 0.23
*Work/community domain*	− 0.59	0.250	− 2.35, 1.17

*M*_*diff*_ mean difference between the groups of changes from pre to post, *AIMS* Arthritis Impact Measurement Scale, *PF* physical functioning, *RAPA* Rapid Assessment of Physical Activity, *AAQ-II* Acceptance and Action Questionnaire, *BEVS* Bull’s Eye Values Survey

**Significant finding at *p* < 0.05

*Approaching significance at *p* < 0.05

#### Daily steps

Objective measures of daily steps were not available. Daily step logs, written by participants were available for a subset of participants (*n* = 25 out of 40, 37% missing data). Daily step logs contained one count of total steps per day based on each participant’s Garmin device step count before bedtime, for seven consecutive days in the baseline assessment period and seven consecutive days in the post-treatment assessment period, and a sum score of the total steps was created for each time point. After examining step logs, there was an additional 5% missing data due to participants not logging daily step counts on some of the days in the assessment periods, for a total of 43% missing data. Despite these high levels of missing data, given that Engage-PA was designed to increase participants’ walking behavior, an analysis of available data was conducted to examine if there was any signal of improvement in this important metric. A repeated measures analysis of variance (ANOVA) was conducted on complete daily steps data (*n* = 18 participants, 9 per group). The results showed there were no significant effects for time (*F* (1,16) = 2.45, *p* = 0.137), group (*F*(1,16) = 0.152, *p* = 0.702), nor was there a time by group interaction (*F*(1,16) = 0.678, *p* = 0.422). Group means are presented in Tables 3 and 5 illustrates within and between-group ANOVA results.

**Table 5:** Within- and between-group ANOVA results for daily steps

	Degrees of freedom	Mean square	***F***	***P***
Between-group	1.15	62,254,766	0.246	0.627
Within-group	1.15	338,207,749	1.381	0.258
Time × condition interaction	1.15	203,506	0.005	0.947

*p* significance value at *p* < 0.05

## Discussion

This is the first study to examine novel, brief, combined values and activity-pacing intervention for improving arthritis-related pain and functioning and increasing physical activity in a sample of older adults with osteoarthritis pain. Engage-PA demonstrated high levels of feasibility and acceptability, and there were indications of improvement in secondary outcomes of arthritis pain and functioning, as well as self-reported physical activity.

It is notable that Engage-PA was feasible even though the study was conducted entirely during the COVID-19 pandemic, especially because most participants reported that the pandemic had a negative impact on general well-being and physical activity. Most had less access to locations to exercise or social support for exercising and were more sedentary due to the pandemic, and 41% reported severe impact from COVID on their health, finances, or other life areas. This indicates that they had more barriers to overcome in order to engage in physical activity than others with OA pain in studies conducted prior to the COVID-19 pandemic. Yet, despite the significant pandemic-related problems with exercising, participants in Engage-PA were recruited, enrolled, retained, and completed sessions at high rates comparable to studies conducted under more ideal circumstances. This may be related to participant feedback that Engage-PA provided not only important motivation and pacing skills for increasing physical activity, but also additional support, problem-solving, and accountability for meeting physical activity goals. Given that older adults with OA pain have long reported significant challenges with initiating and maintaining physical activity, these findings during the COVID-19 pandemic suggest Engage-PA would be feasible and acceptable in the future as well.

Engage-PA shows particular promise for larger-scale implementation. The study was successful in recruiting participants from diverse backgrounds in line with the demographics of patients served in the treating clinics. It is a brief protocol, delivered by a master’s level behavioral interventionist, with many formalized elements (i.e., manual, workbook, personal fitness tracker); features that may be appealing for primary care clinic dissemination.

Finally, Engage-PA was delivered with completely remote procedures, including telephone and online recruitment and enrollment, telephone session delivery, and remote assessment completion (online, telephone-collected, or mailed paper packets). Although there were some challenges related to the fitness-tracker devices, participants largely enjoyed having personalized daily steps feedback.

## Limitations

There are limitations in this study. It was a small feasibility and acceptability pilot trial, not powered to detect effects, and future studies should be adequately powered to detect any intervention effects on primary outcomes of arthritis pain and physical activity. This study was also conducted earlier in the COVID-19 pandemic, which provided additional challenges to the study, and prevented the capture of objective daily steps measurement. While accelerometers are considered the gold standard for objective assessment of physical activity, turning to all-remote procedures made the use of these devices for all participants untenable. Likewise, there were challenges with the Garmin devices used in this study, which required on-site syncing to gather data. We mitigated this challenge by switching to participant-written daily step logs from Garmin step count readings midway through the trial. The non-significant findings for changes in daily steps over time or between the groups were not surprising given the high levels of missingness, but were in contrast to the finding that self-reported physical activity improved over time. It is likely that the daily steps data was less robust and reliable as a result of these pandemic-related challenges with data collection. Future research will be needed to assess the potential of Engage-PA to change daily steps in meaningful ways.

Additionally, participants received some financial benefit for participating in the program; something that is not part of routine clinical practice. Although they were not compensated for enrollment or participating in any intervention sessions, participants received a relatively low-cost fitness tracker (approximately $100/device) for free and were compensated for completing research assessments.

Finally, the participants in this study were mostly women, living independently, and were recruited through a large network of academic medical center-affiliated primary care clinics, and as such may not be fully representative of the population of older adults with arthritis pain and difficulties with walking. Future studies assessing effectiveness should consider recruitment to reach a broader sample that includes those who may be medically underserved, historically marginalized, or living in rural areas.

## Considerations for protocol refinement

Feasibility data was promising for most metrics, but the accelerometers were less feasible in this study, and the personal fitness trackers were not amenable to remote procedures. Some remote procedures for accelerometer use may be feasible in the future, such as mailing accelerometers to participants and providing detailed instructions for correct wear. However, the demands of using study-owned accelerometers (designed for reuse over time) are likely best managed when study staff can be on-site to track when devices are mailed out, sanitize returned devices, and download the data before mailing them out again.

Client Satisfaction Questionnaire data indicated that the session content (e.g., behavioral skills) was interesting and helpful, but most of the challenges reported by participants were related to understanding the interface of the personal fitness trackers. Additionally, the self-report measure of physical activity captured other forms of daily activity such as swimming and strength training, and there were some indicators of improvement in overall activity (not just walking). While walking is an easily accessible activity for many (e.g., timed bouts of walking can be done inside even in small spaces, when poor weather or unsafe neighborhoods make outdoor exercise difficult), future research may consider utilizing personal fitness trackers that can also capture other forms of activity. Furthermore, the strategic activity pacing skill may be applied to help participants engage in other forms of physical activity as well as walking when that is desirable for participants. As such, future iterations of the Engage-PA protocol will likely utilize different fitness tracker devices to reduce data collection problems, increase participant useability and increase functionality.

Additional protocol refinements may be required after learning more in future studies, as data from secondary analyses should be interpreted with extreme caution due to the small sample size and missingness related to daily steps data.

## Conclusions

Engage-PA is a novel, brief behavioral intervention for older adults with OA pain that demonstrates high feasibility and acceptability even when patients present with considerable barriers to establishing physical activity routines (e.g., during the COVID-19 pandemic). Since feasibility and acceptability criteria were met but some features of the protocol need refinement, the next steps should include refinement of protocol based on participant feedback followed by larger effectiveness studies in real-world clinical care settings. If found effective in future studies, Engage-PA may provide important opportunities for older adults to reduce arthritis-related pain, increase physical activity and improve day-to-day function.

## Data Availability

The datasets used and/or analyzed during the current study are available from the corresponding author on reasonable request.

## Abbreviations

OA: Osteoarthritis
ACT: Acceptance and commitment therapy
PCST: Pain coping skills training
COVID-19: Coronavirus disease of 2019
CIS: COVID-19 Impact Scale
Engage-PA: Engage Protocol, Physical Activity Adaptation for OA-Related Pain
TAU+: Treatment-as-usual plus fitness tracker
AIMS: Arthritis Impact Measurement Scale
AAQ-II: Acceptance and Action Questionnaire-II
CSQ: Client Satisfaction Questionnaire
RAPA: Rapid Assessment of Physical Activity
PARQ-2020: Physical Activity Readiness Questionnaire Plus
REDCap: Research Electronic Data Capture
HIPAA: Health Insurance Portability and Accountability Act of 1996
SPSS: Statistical Package for the Social Sciences
CONSORT: Consolidated Standards of Reporting Trials
COPD: Chronic obstructive pulmonary disease
*M*_diff_: Mean difference
CI: Confidence interval
BEVS: Bull’s Eye Values Survey

## References

[1] WHO | Information sheet: global recommendations on physical activity for health 65 years and above. WHO. Available from: https://www.who.int/dietphysicalactivity/publications/recommendations65yearsold/en/. Cited 2020 Jan 28.

[2] Felson DT (1993). The course of osteoarthritis and factors that affect it. Rheum Dis Clin North Am.

[3] Marshall K, Hale D (2019). Older adults and the opioid crisis. Home Healthc Now.

[4] Physical activity helps arthritis pain | CDC. 2019. Available from: https://www.cdc.gov/arthritis/communications/features/physical-activity-helps-arthritis.htm. Cited 2020 Sep 16.

[5] Pahor M, Guralnik JM, Ambrosius WT, Blair S, Bonds DE, Church TS (2014). Effect of structured physical activity on prevention of major mobility disability in older adults: the LIFE study randomized clinical trial. JAMA.

[6] Uthman OA, van der Windt DA, Jordan JL, Dziedzic KS, Healey EL, Peat GM, et al. Exercise for lower limb osteoarthritis: systematic review incorporating trial sequential analysis and network meta-analysis. BMJ. 2013;347 Available from: https://www.bmj.com/content/347/bmj.f5555. Cited 2020 Sep 16. 10.1136/bmj.f5555 PMC3779121 24055922

[7] Singh MAF (2002). Exercise comes of age: rationale and recommendations for a geriatric exercise prescription. J Gerontol A Biol Sci Med Sci.

[8] Physical activity fundamental to preventing disease. ASPE. 2015. Available from: https://aspe.hhs.gov/basic-report/physical-activity-fundamental-preventing-disease. Cited 2020 Jan 28.

[9] Kanavaki AM, Rushton A, Efstathiou N, Alrushud A, Klocke R, Abhishek A, et al. Barriers and facilitators of physical activity in knee and hip osteoarthritis: a systematic review of qualitative evidence. BMJ Open. 2017;7(12) Available from: https://www.ncbi.nlm.nih.gov/pmc/articles/PMC5770915/. Cited 2020 Jan 28. 10.1136/bmjopen-2017-017042 PMC5770915 29282257

[10] Keefe FJ, Kashikar-Zuck S, Opiteck J, Hage E, Dalrymple L, Blumenthal JA (1996). Pain in arthritis and musculoskeletal disorders: the role of coping skills training and exercise interventions. J Orthop Sports Phys Ther.

[11] Somers TJ, Blumenthal JA, Guilak F, Kraus VB, Schmitt DO, Babyak MA (2012). Pain coping skills training and lifestyle behavioral weight management in patients with knee osteoarthritis: a randomized controlled study. Pain.

[12] Barreto M, Gaynor ST (2019). A single-session of acceptance and commitment therapy for health-related behavior change: protocol description and initial case examples. Behav Anal Res Pract.

[13] Kwasnicka D, Dombrowski SU, White M, Sniehotta F (2016). Theoretical explanations for maintenance of behaviour change: a systematic review of behaviour theories. Health Psychol Rev.

[14] Rahal GM, Gon MCC (2020). A systematic review of values interventions in acceptance and commitment therapy. Int J Psychol Psychol Ther.

[15] Hayes SC, Strosahl KD, Wilson KG. Acceptance and commitment therapy, second edition: the process and practice of mindful change. Oakland: Guilford Press; 2011. p. 417.

[16] Zhang C-Q, Leeming E, Smith P, Chung P-K, Hagger MS, Hayes SC (2018). Acceptance and commitment therapy for health behavior change: a contextually-driven approach. Front Psychol.

[17] Hayes SC, Levin ME, Plumb-Vilardaga J, Villatte JL, Pistorello J (2013). Acceptance and commitment therapy and contextual behavioral science: examining the progress of a distinctive model of behavioral and cognitive therapy. Behav Ther.

[18] Plumb JC, Stewart I, Dahl J, Lundgren T (2009). In search of meaning: values in modern clinical behavior analysis. Behav Anal.

[19] Bricker JB, Mull K, Kientz JA, Vilardaga RM, Mercer LD, Akioka K (2014). Randomized, controlled pilot trial of a smartphone app for smoking cessation using acceptance and commitment therapy. Drug Alcohol Depend.

[20] Lillis J, Kendra KE (2014). Acceptance and commitment therapy for weight control: model, evidence, and future directions. J Contextual Behav Sci.

[21] Butryn ML, Forman E, Hoffman K, Shaw J, Juarascio A (2011). A pilot study of acceptance and commitment therapy for promotion of physical activity. J Phys Act Health.

[22] VanBuskirk K, Roesch S, Afari N, Wetherell JL (2014). Physical activity of patients with chronic pain receiving acceptance and commitment therapy or cognitive behavioural therapy. Behav Change.

[23] Vowles KE, McCracken LM, O’Brien JZ (2011). Acceptance and values-based action in chronic pain: a three-year follow-up analysis of treatment effectiveness and process. Behav Res Ther.

[24] Keefe FJ, Abernethy AP, Campbell LC (2005). Psychological approaches to understanding and treating disease-related pain. Annu Rev Psychol.

[25] Somers TJ, Abernethy AP, Edmond SN, Kelleher SA, Wren AA, Samsa GP (2015). A pilot study of a mobile health pain coping skills training protocol for patients with persistent cancer pain. J Pain Symptom Manage.

[26] Somers TJ, Kelleher SA, Westbrook KW, Kimmick GG, Shelby RA, Abernethy AP (2016). A small randomized controlled pilot trial comparing mobile and traditional pain coping skills training protocols for cancer patients with pain. Pain Res Treat.

[27] O’Sullivan ML, Shelby RA, Dorfman CS, Kelleher SA, Fisher HM, Rowe Nichols KA (2018). The effect of pre-transplant pain and chronic disease self-efficacy on quality of life domains in the year following hematopoietic stem cell transplantation. Support Care Cancer.

[28] Kelleher SA, Fisher HM, Winger JG, Somers TJ, Uronis HE, Wright AN (2021). Feasibility, engagement, and acceptability of a behavioral pain management intervention for colorectal cancer survivors with pain and psychological distress: data from a pilot randomized controlled trial. Support Care Cancer.

[29] Dorfman CS, Kelleher SA, Winger JG, Shelby RA, Thorn BE, Sutton LM (2019). Development and pilot testing of an mHealth behavioral cancer pain protocol for medically underserved communities. J Psychosoc Oncol.

[30] Wang L, Zhang L, Yang L, Cheng-qi H (2021). Effectiveness of pain coping skills training on pain, physical function, and psychological outcomes in patients with osteoarthritis: a systemic review and meta-analysis. Clin Rehabil.

[31] Allen K, Somers T, Campbell L, Arbeeva L, Coffman C, Cené C (2019). Pain coping skills training for African Americans with osteoarthritis: results of a randomized controlled trial. Pain.

[32] Bandura A (2001). Social cognitive theory: an agentic perspective. Annu Rev Psychol.

[33] Teo I, Vilardaga JP, Tan YP, Winger J, Cheung YB, Yang GM (2020). A feasible and acceptable multicultural psychosocial intervention targeting symptom management in the context of advanced breast cancer. Psychooncology.

[34] Plumb Vilardaga JC, Winger JG, Teo I, Owen L, Sutton LM, Keefe FJ (2020). Coping skills training and acceptance and commitment therapy for symptom management: feasibility and acceptability of a brief telephone-delivered protocol for patients with advanced cancer. J Pain Symptom Manag.

[35] Warburton DER, Jamnik V, Bredin SSD, Shephard RJ, Gledhill N (2019). The 2020 Physical Activity Readiness Questionnaire for Everyone (PAR-Q+) and electronic Physical Activity Readiness Medical Examination (ePARmed-X+). Health Fit J Can.

[36] Attkisson CC, Zwick R (1982). The client satisfaction questionnaire. Psychometric properties and correlations with service utilization and psychotherapy outcome. Eval Program Plann.

[37] Attkisson CC, Greenfield TK (2004). The UCSF Client Satisfaction Scales: I. The Client Satisfaction Questionnaire-8. The use of psychological testing for treatment planning and outcomes assessment: instruments for adults.

[38] Meenan RF, Mason JH, Anderson JJ, Guccione AA, Kazis LE (1992). AIMS2. The content and properties of a revised and expanded Arthritis Impact Measurement Scales Health Status Questionnaire. Arthritis Rheum.

[39] Topolski TD, LeGerfo J, Patrick DL, Williams B, Walwick J, Patrick MB. The Rapid Assessment of Physical Activity (RAPA) among older adults. Prev Chronic Dis. 2006; [serial online]. Available from: http://www.cdc.gov/pcd/issues/2006/oct/06_001.htm. PMC1779282 16978493

[40] Bond FW, Hayes SC, Baer RA, Carpenter KM, Guenole N, Orcutt HK (2011). Preliminary Psychometric Properties of the Acceptance and Action Questionnaire–II: a revised measure of psychological inflexibility and experiential avoidance. Behav Ther.

[41] Hayes SC, Luoma JB, Bond FW, Masuda A, Lillis J (2006). Acceptance and commitment therapy: model, processes and outcomes. Behav Res Ther.

[42] Graham CD, Gouick J, Krahé C, Gillanders D (2016). A systematic review of the use of acceptance and commitment therapy (ACT) in chronic disease and long-term conditions. Clin Psychol Rev.

[43] Lundgren T, Luoma JB, Dahl J, Strosahl K, Melin L (2012). The Bull’s-Eye Values Survey: a psychometric evaluation. Cogn Behav Pract.

[44] Lundgren T, Dahl J, Hayes SC (2008). Evaluation of mediators of change in the treatment of epilepsy with acceptance and commitment therapy. J Behav Med.

[45] Stoddard J, Kaufman J (2020). Coronavirus impact scale.

[46] Eldridge SM, Chan CL, Campbell MJ, Bond CM, Hopewell S, Thabane L (2016). CONSORT 2010 statement: extension to randomised pilot and feasibility trials. BMJ.

[47] Leon AC, Davis LL, Kraemer HC (2011). The role and interpretation of pilot studies in clinical research. J Psychiatr Res.

